# Mental Illness Following Physical Assault Among Children

**DOI:** 10.1001/jamanetworkopen.2023.29172

**Published:** 2023-08-16

**Authors:** Étienne Archambault, Simone N. Vigod, Hilary K. Brown, Hong Lu, Kinwah Fung, Michelle Shouldice, Natasha Ruth Saunders

**Affiliations:** 1 Institute for Health Policy, Management and Evaluation, University of Toronto, Toronto, Ontario, Canada; 2 ICES, Toronto, Ontario, Canada; 3 Centre Hospitalier Universitaire (CHU) Sainte-Justine, Montréal, Quebec, Canada; 4 Women’s College Research Institute, Women’s College Hospital, Toronto, Ontario, Canada; 5 Department of Psychiatry, University of Toronto, Toronto, Ontario, Canada; 6 Interdisciplinary Centre for Health and Society, University of Toronto Scarborough, Scarborough, Ontario, Canada; 7 Dalla Lana School of Public Health, University of Toronto, Toronto, Ontario, Canada; 8 Department of Pediatrics, University of Toronto, Toronto, Ontario, Canada; 9 Division of Pediatric Medicine, The Hospital for Sick Children, Toronto, Ontario, Canada; 10 Child Health Evaluative Sciences, SickKids Research Institute, Toronto, Ontario, Canada

## Abstract

**Importance:**

Physical assault during childhood is common and can lead to lasting mental health problems. Yet, there are few studies on the patterns of mental illness (ie, timing of onset, type, and acuity) in survivors of physical assault.

**Objective:**

To determine the risk of incident health record diagnoses of mental illness among children who experienced assault compared with children who did not.

**Design, Setting, and Participants:**

This population-based matched cohort study used linked health administrative data sets in Ontario, Canada. Children aged 0 to 13 years who experienced an incident physical assault between 2006 and 2014 were age-matched (1:4) to children who had not experienced assault and followed up for a minimum of 5 years. Data were analyzed from January 2020 to March 2022.

**Exposure:**

Physical assault resulting in hospitalization or an emergency department (ED) visit between the ages of 0 and 13 years.

**Main Outcomes and Measures:**

The primary outcome was incident health record diagnosis of mental illness measured as any physician or hospital mental health care use or completed suicide. Secondary outcome measures included the acuity of incident mental illness and mental illness diagnostic category. Cox proportional hazards regression analysis generated hazard ratios (HR) for incident mental illness.

**Results:**

A total of 21 948 children unexposed to assault and 5487 exposed to assault were included in the study with a mean (SD) age of 7.0 (4.6) years. There were more boys in the group that experienced assault (3006 individuals [54.8%]) compared with the group who did not (9909 individuals [45.1%]). Compared with children unexposed to assault, those exposed were more likely to be in the highest deprivation index quintile (standardized difference, 0.21) and live in rural areas (standardized difference, 0.48). Their mothers more often had active mental illness (standardized difference, 0.35). More than one-third of the exposed children had a health record diagnosis of mental illness (2219 children [38.6%]; incidence rate (IR), 53.3 per 1000 person-years) compared with 23.4% (5130 children; IR, 32.2 per 1000 person-years) of unexposed children, with an overall adjusted hazard ratio (aHR) of 1.96 (95% CI, 1.85-2.08). The greatest risk was observed in the first year following the assault (aHR, 3.08; 95% CI, 2.68-3.54). In both groups, nonpsychotic disorders were the most common type of mental illness. Initial mental illness diagnoses occurred in an acute care setting for 14.0% of exposed children (769 children) vs 2.8% of unexposed children (609 children).

**Conclusions and Relevance:**

In this population-based matched cohort study, children who experienced assault had, on average, a 2 times higher risk of receiving a mental illness diagnosis and were more likely than children who had not experienced assault to present to acute care for mental illness. Early intervention to support mental health of assaulted children is warranted, particularly in the first year following assault.

## Key Points

**Question:**

Do children and adolescents physically assaulted during childhood have increased risk of mental illness following assault?

**Findings:**

In this matched cohort study of 5487 children who experienced assault during childhood, compared with 21 948 children who did not, the risk of incident mental illness was, on average, 2 times higher among children exposed to physical assault. In the first year following assault, the risk of mental illness in assaulted children was 3 times higher.

**Meaning:**

In this study, physical assault during childhood was associated with an increased risk of mental illness, especially in the first year following assault, suggesting early intervention to support mental health following assault is warranted.

## Teaser

This cohort study evaluates characterizations of mental illness following physical assault among children in Ontario, Canada.

## Introduction

Physical assault of children, be it from a caregiver, a peer, or a stranger, is common across the globe. According to 1 survey study,^1^ more than 50% of US children (aged 0-17 years) experienced a physical assault, and 14.5% of US children experienced an assault resulting in injury in their lifetime. Physical assault during the formative years, either as a solitary event or repeated chronic assaults, may be life-changing, traumatic, or stressful, and is associated with future mental illness, including both internalizing and externalizing disorders.^2,3,4,5,6,7,8^ Studies supporting this association have mostly been cross-sectional in design and primarily conducted in adults self-reporting childhood assault and mental health outcome measures. Patient recall can limit the amount of detail related to physical assault or mental illness diagnosis, especially when these occur at very young ages.^9,10,11^ Furthermore, little is known about the timing of mental illness onset or the type (eg, mood disorders, psychotic disorders, and so forth) and severity of mental illness (eg, the need for acute care) in childhood survivors of physical assault.

Poor mental health in children and youths is greatly associated with the development of future health problems and premature death, as well as important social, economic, and functional impairment.^12,13,14^ Early interventions (eg, school-based programs) have been shown to be effective for many different mental health disorders.^15^ A better understanding of the risks and patterns of mental illness following physical assault is critical for the implementation of timely evidence-based prevention, screening, and treatment strategies. Using a population-based cohort design, the aim of this study was to determine the risk of incident health record diagnoses of mental illness and describe the patterns of these mental illness diagnoses (including the timing of onset, type, and acuity) among children who received acute medical care for physical assault compared with age-matched children who did not experience assault. We hypothesized that children who experienced physical assault would have a high risk of mental illness, particularly in the year following their assault. We also anticipated that childhood survivors of physical assault would present in more acute settings and with both internalizing and externalizing mental health problems.

## Methods

### Study Design and Setting

We conducted a population-based matched cohort study of Ontario children using health administrative data sets that were linked and analyzed at ICES. ICES is an independent, nonprofit research institute whose legal status under Ontario’s health information privacy law allows it to collect and analyze personal health information to conduct approved research projects. In Ontario (population approximately 15 million residents), physician and hospital services are delivered to residents at no direct cost through a single-payer provincial funding system. We conducted this study in accordance with the Reporting of Studies Conducted Using Observational Routinely-Collected Data (RECORD)^16^ and the Strengthening the Reporting of Observational Studies in Epidemiology (STROBE)^17^ reporting guidelines. Research ethics board approval was obtained from the Hospital for Sick Children. Participant consent was waived as data were deidentified.

### Data Sources

Each Ontario resident eligible for health care is assigned a unique and encoded ICES Key Number, which is used to link individual records across a large breadth of databases. We used Ontario’s health system registry data (Registered Persons Database), outpatient physician billing data (Ontario Health Insurance Plan Claims Database), hospital (Discharge Abstract Database), Ontario Mental Health Reporting System and emergency department (National Ambulatory Care Reporting System) discharge data, as well as vital statistics data in our study. Neighborhood characteristics were obtained using Canadian Census data. We also used the MOMBABY database^18^ (ICES-derived database that links newborns delivered in Ontario hospitals to their mothers) to ascertain maternal characteristics and the Immigration, Refugees, and Citizenship Canada Permanent Resident Database^19^ to collect immigrant data. Please refer to eTable 1 in Supplement 1 for additional details on all these data sources.

### Study Population

Our cohort included all children aged 0 to 13 years living in Ontario, Canada, with an incident physical assault requiring acute medical care (index event) between April 1, 2006, and March 31, 2014. Children were excluded if they died during the index emergency department (ED) visit or hospitalization for physical assault, if there was a simultaneous diagnosis of intentional self-injury on the index event date, if they had an invalid ICES Key Number, were non-Ontario residents, or had a missing age, sex, or birthdate in the Registered Persons database. Children were also excluded from the cohort if they had a history of mental illness before the index event date. Included children were then matched to 4 (1:4) unexposed children. Matching was conducted according to age (±1 month) and maternal linkage (ie, the ability to link children to their mothers via the MOMBABY database). Limiting the number of matching criteria permitted a more in-depth analysis of the differences between groups as well as the association between many different covariate physical assault and mental health outcomes. Children were followed until their 18th birthday or until study end date (March 31, 2019), allowing for at least 5 years of follow-up for each child. Please refer to the eFigure in Supplement 1 for the study flowchart.

### Exposure

The exposure was incident physical assault resulting in an ED visit or hospitalization. Physical assault occurs when a child is physically harmed or at substantial risk of experiencing physical harm because of an abusive act.^3^ Assault was ascertained from the discharge diagnoses of ED and hospital records using validated^20,21^ *International Classification of Diseases 10th Revision* (*ICD-10-CA*)^22^ codes (eTable 2 in Supplement 1). Data on perpetrators of the assault were not available in existing data sets.

### Covariates

According to a review of the literature, clinical plausibility, and/or data availability, several baseline characteristics and other covariates were included in the current study given their association with both exposure and outcome (ie, potential confounding factors). Baseline characteristics were measured at the index event date and included age, sex, socioeconomic status (measured as 2 categorical variables: neighborhood income quintile and neighborhood-level material deprivation index quintile), rurality, and immigration status. Other covariates included exposure to other forms of child maltreatment (eTable 3 in Supplement 1), as well as maternal variables (eTable 4 in Supplement 1) for children born in Ontario, which included maternal age at birth, maternal active mental illness (diagnosis in the 2 years before the index event date), maternal exposure to violence resulting in assessment by a health care clinician in an acute setting, and health record documentation of maternal marital problems or divorce. Existing data sets do not have paternal linkage available.

### Outcomes

The primary outcome was incident health record diagnosis of mental illness measured as any physician or hospital mental health care use (outpatient, ED, or hospitalization) or completed suicide. Outpatient physician visits were identified using a validated algorithm for ambulatory mental health care modified by adding specific pediatric diagnostic and fee codes^23,24,25^ (eTable 5 in Supplement 1). To reduce the likelihood of misclassification bias, a 2-month washout period (period during which mental outcomes were excluded from analysis) was implemented for outpatient mental health outcomes. *ICD-10-CA*^22^ and *ICD-9-CM*^26^ codes were used to identify mental illness from discharge diagnoses of ED and hospitalization records (eTable 5 in Supplement 1). Suicide-related deaths were ascertained using the vital statistics death registry.^27^

Secondary outcomes included (1) the acuity of incident mental illness (ie, presentation to an ED or hospital vs an outpatient mental illness visit), and (2) the diagnostic category of initial mental illness diagnosis. Mental illness diagnostic categories included psychotic disorders, nonpsychotic disorders, substance use disorders, select childhood behavior disorders, intentional self-injury, and other disorders (eTable 5 in Supplement 1).

### Statistical Analyses

We used descriptive statistics to summarize the exposure variable, primary and secondary outcome measures, as well as all covariates for both the children who experienced assault and those who did not. Given the anticipated large sample size, differences between groups were assessed using standardized differences (standardized differences greater than 0.10 were considered clinically meaningful).^28^ Incident rates (IRs) per 1000 person-years were also calculated for the primary outcome measure.

Survival analysis was used to study the occurrence and timing of incident mental illness. The Kaplan-Meier estimator generated the time to incident health record diagnosis of mental illness survival curve. We conducted a Cox proportional hazards regression analysis to examine the association between physical assault and incident mental illness. The association between physical assault on incident mental illness was summarized using crude and adjusted hazard ratios displayed with a 95% CI. We evaluated the proportional hazards assumption of the Cox proportional hazards regression models for all included explanatory variables. Nonproportionality was handled by partitioning the time axis (which provided us with a better understanding of the timing of mental illness diagnosis) and/or including time-dependent covariates in the regression model.^29^ To further assess the robustness of study results, regression models were also generated using the individual mental illness diagnostic categories as the outcome variables.

The main multivariable models controlled for sex, rurality, material deprivation index quintile, other forms of child maltreatment, and immigrant category. Maternal variables and immigrant category could not be included as confounding variables in the same regression model given maternal variables can only be ascertained for nonimmigrant children (ie, missing data for all immigrant and refugee children). We therefore fitted a second regression model to a restricted cohort of children, which only included children who could be paired to their mothers via the MOMBABY database; maternal variables were additionally controlled for in this model. Because the proportion of missing data for the explanatory variables was expected to be low (<5%), no adjustments were conducted for missing data. All statistical analysis was conducted using SAS Enterprise Guide 7.1 (SAS Institute) from January 2020 to March 2022.

## Results

A total of 27 435 children (mean [SD] age 7.0 [4.6]) years were included in the study cohort, including 5487 children with a physical assault diagnosis in an acute care setting and 21 948 unexposed children. Table 1 details the baseline characteristics of the children included in the study cohort by exposure status. There were more boys (3006 individuals [54.8%]) in the group who experienced assault compared with the group who did not (9909 individuals [45.1%]). Exposed children were also more commonly in the highest material deprivation index quintile (1822 children [33.2%]) compared with unexposed children (5213 children [23.8%]; standardized difference, 0.21). A greater proportion of children who experienced assault lived in rural areas (1217 children [22.2%] compared with 1307 [6.0%] in the group who did not experience assault; standardized difference, 0.48). Children who experienced assault were more frequently nonimmigrants (5231 children [95.3%] compared with 19 871 [90.5%] in the unexposed group). There was also a smaller proportion of nonrefugee immigrants in the group who experienced assault (159 children [2.9%] compared with 1694 [7.7%] in the group who did not experience assault). The proportion of refugees was similar in both groups. The median (IQR) follow-up time was 6.79 (5.30-9.14) years in the exposed group and 6.84 (5.32-9.18) years in the unexposed group. More than one-third of mothers in the exposed group (1894 individuals [34.5%]) had active mental illness compared with 4195 mothers in the unexposed group (19.1%) (standardized difference, 0.35). Moreover, a higher proportion of mothers in the exposed group had a health record of marital problems or divorce (186 individuals [3.4%] vs 373 individuals [1.7%]) as well as a history of physical or sexual assault (250 individuals [4.6%] vs 100 individuals [0.5%]) for which they sought care. Moreover, 760 mothers in the group who experienced assault (13.9%) were younger than 19 years at the time they gave birth to the child included in the study cohort (vs 423 mothers [1.9%)] in the group who did not experience assault).

**Table 1.** Baseline Characteristics of Childhood Survivors of Physical Assault and Matched Unexposed Children

Baseline characteristic	Children, No. (%)		
	Unexposed to assault (n = 21 948)	Exposed to assault (n = 5487)	Standardized difference^a^
Age (at index), mean (SD), y	7.03 (4.6)	7.03 (4.6)	0.00
Sex			
Male	9909 (45.1)	3006 (54.8)	0.19
Female	12 039 (54.9)	2481 (45.2)	
Material Deprivation Index			
Quintile 1	3788 (17.3)	615 (11.2)	0.17
Quintile 2	3890 (17.7)	733 (13.4)	0.12
Quintile 3	3891 (17.7)	952 (17.4)	0.01
Quintile 4	4308 (19.6)	1006 (18.3)	0.03
Quintile 5	5213 (23.8)	1822 (33.2)	0.21
Missing	858 (3.9)	359 (6.5)	0.12
Neighborhood Income			
Quintile 1	4494 (20.5)	1751 (31.9)	0.26
Quintile 2	4025 (18.3)	1175 (21.4)	0.08
Quintile 3	4341 (19.8)	966 (17.6)	0.06
Quintile 4	4576 (20.8)	839 (15.3)	0.14
Quintile 5	4410 (20.1)	678 (12.4)	0.21
Missing	102 (0.5)	78 (1.4)	0.10
Rurality (rural)	1307 (6.0)	1217 (22.2%)	0.48
Immigrant category			
Nonimmigrant	19 871 (90.5)	5231 (95.3)	0.19
Immigrant	1694 (7.7)	159 (2.9)	0.22
Refugee	383 (1.7)	97 (1.8)	0.00
Type of assault			
Sexual assault	NA^b^	16 (0.3)	0.07
Maltreatment other	NA^b^	9 (0.2)	0.06
Parent child problems	NA^b^	28 (0.5)	0.10
Other forms of child maltreatment^c^	NA^b^	52 (0.9)	0.14
Maternal linkage	17 680 (80.6)	4420 (80.6)	0.00
Maternal active mental illness	4196 (19.1)	1894 (34.5)	0.35
Marital problem or divorce	373 (1.7)	186 (3.4)	0.11
Maternal violence or assault	100 (0.5)	250 (4.6)	0.26
Maternal age <19 y at birth	423 (1.9)	760 (13.9)	0.45

Abbreviation: NA, not applicable.

^a^ A standardized difference greater than 0.10 is considered significant.

^b^ Presence of small cells omitted from the table to reduce the risk of reidentification.

^c^ Composite variable composed of sexual assault, maltreatment other, and parent child problems.

### Incident Health Record Diagnosis of Mental Illness

More than one-third of assaulted children (2119 children [38.6%]; IR, 53.28 per 1000 person-years) received a mental health diagnosis according to health records compared with 23.4% of unexposed children (5130 children; IR, 32.16 per 1000 person-years) (Table 2). The Figure shows the Kaplan-Meier time to incident health record diagnosis of mental illness survival curve for children categorized by exposure group. Results from the Cox proportional hazards regression analysis are summarized in Table 3. Exposed children were more likely to have an incident health record diagnosis of mental illness compared with unexposed children (overall crude hazard ratio [cHR], 1.90; 95% CI, 1.80-2.01 and overall adjusted hazard ratio [aHR], 1.96; 95% CI, 1.85-2.08). To account for nonproportionality, the study period was divided into 3 distinct time intervals. Children who experienced assault had the greatest risk of receiving a mental illness diagnosis in the first year following physical assault, with an aHR of 3.08 (95% CI, 2.68-3.54). The higher risk of mental illness persisted over time with an aHR of 2.01 (95% CI, 1.84-2.19) 1 to less than 4 years following physical assault and an aHR of 1.53 (95% CI, 1.39-1.69) 4 or more years following physical assault.

**Table 2.** Mental Health Outcomes in Childhood Survivors of Physical Assault and Matched Unexposed Children

Mental health outcome	Children, No. (%)		
	Unexposed to assault (n = 21 948)	Exposed to assault (n = 5487)	Standardized difference^a^
Incident mental illness	5130 (23.4)	2119 (38.6)	0.33
Acuity of mental illness^b^			
Nonacute outpatient mental illness diagnosis	4531 (20.6)	1459 (26.6)	0.14
Acute care mental illness diagnosis^c^	609 (2.8)	769 (14.0)	0.41
Individual components of the composite mental illness variable^d^			
Nonacute outpatient visit	4991 (22.7)	1952 (35.6)	0.29
Intentional self-injury	103 (0.5)	131 (2.4)	0.16
Emergency department visit	576 (2.6)	727 (13.2)	0.40
Inpatient admission	180 (0.8)	253 (4.6)	0.23
Suicide^e^	NA^f^	NA^f^	0.02
Incident mental illness diagnostic category			
Intentional self-injury	33 (0.2)	42 (0.8)	0.09
Nonpsychotic disorders	2320 (10.6)	890 (16.2)	0.17
Select childhood behavior disorders^g^	1141 (5.2)	544 (9.9)	0.18
Substance use disorders	98 (0.4)	124 (2.3)	0.16
Psychotic disorders	62 (0.3)	27 (0.5)	0.03
Other disorders^h^	1474 (6.7)	490 (8.9)	0.08

Abbrevation: NA, not applicable.

^a^ A standardized difference greater than 0.10 is considered significant.

^b^ Acuity of mental illness at the time of incident mental illness diagnosis (first contact with the health care system).

^c^ Acute care refers to emergency department visit, inpatient admission, or suicide.

^d^ Prevalence throughout the observation period.

^e^ There was a total of 6 suicide-related deaths in both groups combined.

^f^ Presence of small cells omitted from table to reduce the risk of reidentification.

^g^ Attention-deficit/hyperactivity disorder, oppositional defiant disorder and conduct disorder.

^h^ Include eating, sleep, sexual, developmental, intellectual, gender identity, and tic disorders.

**Figure.**
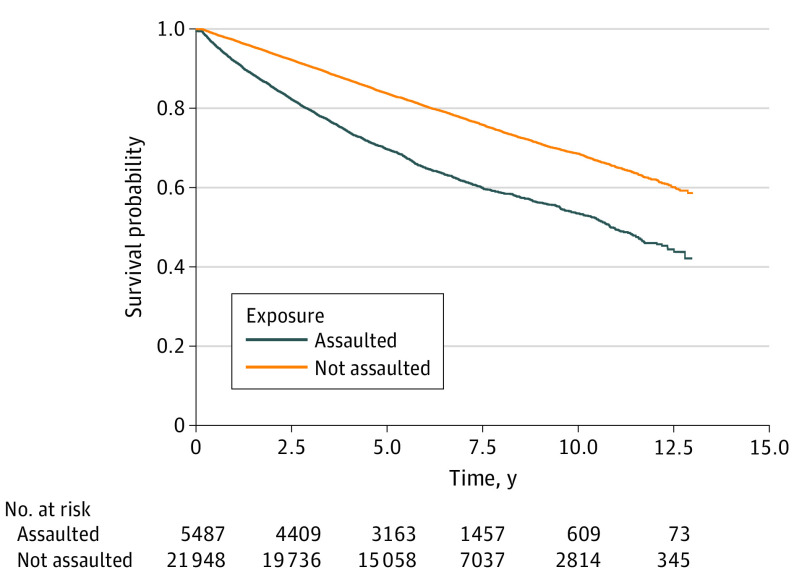
Kaplan-Meier Time to Incident Health Record Diagnosis of Mental Illness Survival Curve

**Table 3.** Association Between Childhood Physical Assault Diagnosed in an Acute Care Setting and Incident Health Record Diagnoses of Mental Illness^a^

Characteristic	No.	Patient-years	No. (%) with mental illness	IR per 1000 person-years (95% CI)	cHR (95% CI)	aHR (95% CI)
Childhood survivors of physical assault	5487	39 769	2119 (38.6)	53.28 (51.04-55.60)	1.90 (1.80-2.01)	1.96 (1.85-2.08)^b^
Unexposed children	21 948	159 537	5130 (23.4)	32.16 (31.28-33.05)	1 [Reference]	1 [Reference]
Time following physical assault diagnosis						
0 to <1 y Following physical assault						
Childhood survivors of physical assault	5487	5457	441 (8.0)	80.81 (73.44-88.71)	3.05 (2.69-3.46)	3.08 (2.68-3.54)^c^
Unexposed children	21 948	21 773	601 (2.7)	27.60 (25.44-29.90)	1 [Reference]	1 [Reference]
1 to <4 y Following physical assault						
Childhood survivors of physical assault	4995	14 794	958 (19.2)	64.76 (60.72-68.99)	1.94 (1.79-2.10)	2.01 (1.84-2.19)^c^
Unexposed children	21 035	62 449	2146 (10.2)	34.36 (32.93-35.85)	1 [Reference]	1 [Reference]
≥4 y Following physical assault						
Childhood survivors of physical assault	3934	13 946	720 (18.3)	51.63 (47.92-55.54)	1.48 (1.35-1.62)	1.53 (1.39-1.69)^b^
Unexposed children	18 535	64 191	2383 (12.9)	37.12 (35.65-38.64)	1 [Reference]	1 [Reference]

Abbreviations: aHR, adjusted hazard ratio; cHR, crude hazard ratio; IR, incidence rate.

^a^ Hazard ratio adjusted for sex, rurality, material deprivation index quintile, other forms of child maltreatment, and immigrant category.

^b^ aHR: Main exposure adjusted for sex, time × sex, rurality, material deprivation index quintile, other forms of child maltreatment, and immigrant category.

^c^ aHR: Main exposure adjusted for sex, rurality, material deprivation index quintile, other forms of child maltreatment, and immigrant category.

### Secondary Mental Health Outcomes

At the time of first contact with the health care system, 14.0% of childhood survivors of physical assault (769 children) received a mental illness diagnosis in an acute care context (ie, ED visit or hospitalization) compared with 2.8% of children who did not experience assault (609 children). The most common incident mental illness diagnostic category for both groups was nonpsychotic disorders (eg, mood and anxiety disorders) (890 exposed children [16.2%]; 2320 unexposed children [10.6%]) with important differences also observed between groups for select childhood behavior disorders (544 exposed children [9.9%]; 1141 unexposed children [5.2%]), and substance use disorders (124 exposed children [2.4%]; 98 unexposed children [0.4%]).

There was a significant association between physical assault and most of the different mental illness diagnostic categories, with substance use disorders (cHR, 17.33; 95% CI, 7.14-42.11) and intentional self-injury (aHR, 8.50; 95% CI, 4.45-16.21) showing the greatest associations in the first year following physical assault (Table 4). Results from the restricted study cohort which excluded children who could not be linked to their mothers via the MOMBABY database were similar to the main study cohort (eTable 6, eTable 7, eTable 8, and eTable 9 in Supplement 1).

**Table 4.** Association Between Childhood Physical Assault Diagnosed in an Acute Care Setting and the Different Mental Illness Diagnostic Categories^a^

Time following physical assault diagnosis	aHR (95% CI)					
	Psychotic disorders	Nonpsychotic disorders	Select childhood behavior disorders^b^	Intentional self-injury	Substance use disorders	Other disorders^c^
0 to <1 y	NA	2.91 (2.26-3.74)^d^	4.03 (3.09-5.27)	NA	17.33 (7.14-42.11)^e^^,^^f^	2.49 (1.97-3.15)
1 to <4 y	NA	2.08 (1.82-2.38)^g^	2.07 (1.73-2.47)	NA	4.83 (2.83-8.27)^h^	1.43 (1.21-1.69)
≥4 y	NA	1.42 (1.24-1.62)	1.81 (1.40-2.34),	5.32 (3.35-8.45)	4.31 (2.53-7.33)	0.86 (0.69-1.07)
0 to <4 y	NA	NA	NA	8.50 (4.45-16.21)	NA	NA
≥0 y	1.92 (1.09-3.39)	NA	NA	NA	NA	NA

Abbreviations: aHR, adjusted hazard ratio; NA, not applicable.

^a^ Hazard ratio adjusted for sex, rurality, material deprivation index quintile, other forms of child maltreatment, and immigrant category.

^b^ Attention deficit/hyperactivity disorder, oppositional defiant disorder and conduct disorder.

^c^ Include eating, sleep, sexual, developmental, intellectual, gender identity, and tic disorders.

^d^ Up to 11 months due to nonproportional hazards.

^e^ Crude hazard ratio presented here because there were few outcomes in that time interval.

^f^ Up to 14 months due to nonproportional hazards.

^g^ 11 months to 4 years due to nonproportional hazards.

^h^ 14 months to 4 years due to nonproportional hazards.

## Discussion

This large population-based matched cohort study demonstrated that children who received a physical assault diagnosis in an acute care setting were more likely to have a health record diagnosis of mental illness following their assault. Although the risk of mental illness was greatest in the first year following assault, the elevated risk of developing a mental illness persisted past the first 4 years following the assault. Assaulted children were more likely to present to acute care for mental health support and a large proportion presented with mood and anxiety disorders.

We observed that the risk of an incident health record diagnosis of mental illness was on average twice as high in children with a physical assault diagnosis in an acute care setting compared with children who did not experience assault. These findings are comparable with existing data using self-report. Cheung et al^7^ observed that the odds of mental illness were 3.4 times higher (99% CI, 2.4-4.8) in adolescents who reported physical abuse compared with those who did not. In another study conducted in adolescents, the odds of internalizing and externalizing behavior were, respectively, 1.76 times and 2.29 times higher in youths who were investigated by a child protection agency for physical abuse compared with youths with no such history.^30^ Although it was expected that children who experienced assault would have a higher risk of mental illness, a novel finding from our study is that the risk was highest in the first year following physical assault (aHR, 3.08), persisting for more than 4 years following the assault.

Compared with children who were not physically assaulted, children in the assault group more often presented to an acute care setting (ED visit or hospitalization) with their first mental illness presentation. Although the setting or acuity of incident mental illness in children following physical assault has not been well described in the literature, our findings could mean that childhood survivors of physical assault have a more severe presentation of mental illness or present more often in the context of a mental health crisis. It is also possible that those experiencing assault seek care more frequently in an acute care setting because of the difficult access to timely outpatient mental health care in this more vulnerable population of children and youths.^31,32,33^ In our study, we also observed that mothers of children who experienced assault were more likely to have an active mental illness and present with factors associated with family stressors (eg, a history of divorce or exposure to violence) which have been shown to be associated with an increased risk of child maltreatment (including physical assault) and mental illness in children.^3,34,35,36,37,38,39^

From a clinical and policy perspective, our study highlights that there is a critical opportunity for health care clinicians to support children in the first year following physical assault. There is a need to develop and implement targeted mental illness prevention, screening, and treatment programs for assaulted children, which should be the subject of future research studies. Our results also advocate for accessible mental health care outside of the acute setting and for care that addresses the social and health needs of mothers, who themselves have high social and health risks.

### Limitations

This study has a number of limitations. Given that child maltreatment commonly goes undetected and that many forms of maltreatment will not prompt a visit to an ED or a hospitalization, the number of children included in our study is likely an underestimate of the true scope of childhood physical assault. Mental health outcomes were also underestimated because our databases did not capture children and youths with mental health problems who did not engage with the health care system and those who received care from a nonhospital social worker or psychologist as these clinicians’ data are not available in existing data sets. Nearly half of all children with an acute care diagnosis of physical assault between 2006 and 2014 were excluded from our study because they had a history of mental illness. Prior mental illness is an important confounding factor of the association between physical assault and mental illness, which is why we only included children in our study if they had no history of mental illness at the time of physical assault diagnosis. Excluding those children has most likely led to an underestimation of the true scope of mental health problems in childhood survivors of physical assault. However, from a clinical perspective, irrespective of prior mental illness, our results most likely remain valid and applicable for any child presenting in an acute care setting for a physical assault.

Research has shown that certain individual factors, such as resiliency and other family or community supports, may be protective and can influence how a child may respond to violence.^7,40,41^ Other factors, such as certain perpetrator characteristics (eg, caregiver assault vs school bullying), may precipitate negative mental health outcomes following assault^5^ and impact which children ultimately present to care. The effect of such factors could not be explored in our study, which is an important limitation in our attempt to better understand the pathway to mental illness following physical assault.

Although we used many data sets for this study, there were several potential unmeasured confounders. These include paternal covariates (existing databases do not allow linkage between children and their fathers or other caregivers) as well as other adverse childhood experiences, such as parental incarceration or neighborhood violence. Residual confounding, either from unmeasured or unknown confounders, could affect the validity of our regression models and the resulting measures of risk.

It is also possible that children who experienced assault more frequently encounter the health system due to their injury and have greater opportunity for mental illness detection. This was partially mitigated by using a washout period for mental illness identification following the index injury. Despite this potential ascertainment bias, our findings and strength of association persisted well after the initial injury and its sequela, and routine screening for mental illness is not performed in most health care settings for children in Ontario.

## Conclusions

In this study, children with a physical assault diagnosis in an acute care setting were at elevated risk of receiving a mental illness diagnosis according to health records, particularly in the first year following physical assault. These findings highlight that there may be a critical opportunity to support children, and potentially their mothers, in the period immediately following their assault. It will also be important to tailor intervention programs toward the more prevalent types of mental illness diagnosed in childhood survivors of physical assault including nonpsychotic disorders and childhood behavior disorders. Our study also shows that better accessibility to mental health community supports, including through primary care, may be warranted to prevent mental health crises.

## Notes

Supplement 1. **eFigure.** Study Cohort Flow Chart **eTable 1.** Data Sources and Variables Ascertained **eTable 2.** *ICD-10-CA* Codes Used to Define Physical Assault **eTable 3.** *ICD-10-CA* and OHIP Billing Codes Used to Define Sexual Assault and Other Forms of Child Maltreatment **eTable 4.** *ICD-10-CA* and OHIP Billing Codes Used to Define Maternal Variables **eTable 5.** Mental Illness Codes and Diagnostic Categories Used to Define Primary and Secondary Mental Health Outcomes **eTable 6.** Baseline Characteristics of Childhood Survivors of Physical Assault and Matched Unexposed Children (Cohort Restricted to Children Linked to Their Mothers via the MOMBABY Database) **eTable 7.** Association Between Childhood Physical Assault Diagnosed in an Acute Care Setting and Incident Health Record Diagnoses of Mental Illness in the Restricted Study Cohort: Incidence Rate as Well as Crude and Adjusted Hazard Ratios **eTable 8.** Mental Health Outcomes in Childhood Survivors of Physical Assault and Matched Unexposed Children (Cohort Restricted to Children Linked to Their Mothers via the MOMBABY Database) **eTable 9.** Association Between Childhood Physical Assault Diagnosed in an Acute Care Setting and the Different Mental Illness Diagnostic Categories in the Restricted Study Cohort: Adjusted Hazard Ratios

Supplement 2.
Data Sharing Statement
