# Supplementary material for: Mental Illness Following Physical Assault Among Children
Source: JAMA Netw Open. 2023 Aug 16;6(8):e2329172. doi: 10.1001/jamanetworkopen.2023.29172 (PMC10433085; doi:10.1001/jamanetworkopen.2023.29172)
Supplement: Supplement 1. — eFigure. Study Cohort Flow Chart eTable 1. Data Sources and Variables Ascertained eTable 2. ICD-10-CA Codes Used to Define Physical Assault eTable 3. ICD-10-CA and OHIP Billing Codes Used to Define Sexual Assault and Other Forms of Child Maltreatment eTable 4. ICD-10-CA and OHIP Billing Codes Used to Define Maternal Variables eTable 5. Mental Illness Codes and Diagnostic Categories Used to Define Primary and Secondary Mental Health Outcomes eTable 6. Baseline Characteristics of Childhood Survivors of Physical Assault and Matched Unexposed Children (Cohort Restricted to Children Linked to Their Mothers via the MOMBABY Database) eTable 7. Association Between Childhood Physical Assault Diagnosed in an Acute Care Setting and Incident Health Record Diagnoses of Mental Illness in the Restricted Study Cohort: Incidence Rate as Well as Crude and Adjusted Hazard Ratios eTable 8. Mental Health Outcomes in Childhood Survivors of Physical Assault and Matched Unexposed Children (Cohort Restricted to Children Linked to Their Mothers via the MOMBABY Database) eTable 9. Association Between Childhood Physical Assault Diagnosed in an Acute Care Setting and the Different Mental Illness Diagnostic Categories in the Restricted Study Cohort: Adjusted Hazard Ratios [file jamanetwopen-e2329172-s001.pdf]

Supplemental Online Content

Archambault É, Vigod SN, Brown HK, et al. Mental illness following physical assault among children. *JAMA Netw Open*. 2023;6(8):e2329172. doi:10.1001/jamanetworkopen.2023.29172

eFigure. Study Cohort Flow Chart

eTable 1. Data Sources and Variables Ascertained

eTable 2. *ICD-10-CA* Codes Used to Define Physical Assault

eTable 3. *ICD-10-CA* and OHIP Billing Codes Used to Define Sexual Assault and Other Forms of Child Maltreatment

eTable 4. *ICD-10-CA* and OHIP Billing Codes Used to Define Maternal Variables

eTable 5. Mental Illness Codes and Diagnostic Categories Used to Define Primary and Secondary Mental Health Outcomes

eTable 6. Baseline Characteristics of Childhood Survivors of Physical Assault and Matched Unexposed Children (Cohort Restricted to Children Linked to Their Mothers via the MOMBABY Database)

eTable 7. Association Between Childhood Physical Assault Diagnosed in an Acute Care Setting and Incident Health Record Diagnoses of Mental Illness in the Restricted Study Cohort: Incidence Rate as Well as Crude and Adjusted Hazard Ratios

eTable 8. Mental Health Outcomes in Childhood Survivors of Physical Assault and Matched Unexposed Children (Cohort Restricted to Children Linked to Their Mothers via the MOMBABY Database)

eTable 9. Association Between Childhood Physical Assault Diagnosed in an Acute Care Setting and the Different Mental Illness Diagnostic Categories in the Restricted Study Cohort: Adjusted Hazard Ratios

This supplemental material has been provided by the authors to give readers additional information about their work.

© 2023 Archambault É et al. *JAMA Network Open.*

eFigure. Study Cohort Flow Chart

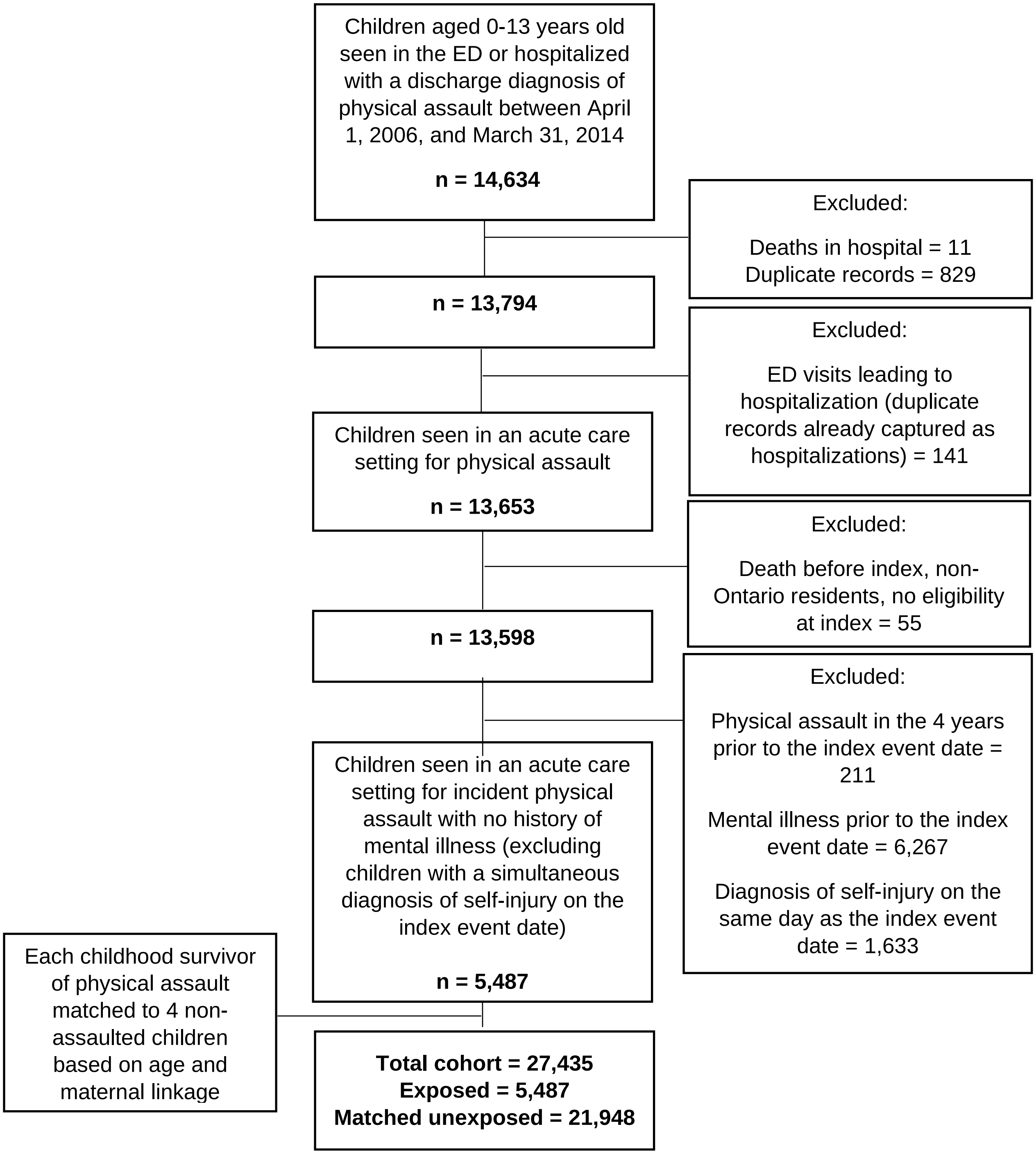

ED = Emergency department

© 2023 Archambault É et al. *JAMA Network Open.*

eTable 1. Data Sources and Variables Ascertained

Data source	Variables ascertained	Characteristics
Registered Persons Database (RPDB)	Age and sex	• Contains demographic information about all individuals eligible for provincial health insurance in Ontario.<br>• Data have been collected and reported since 1991.
Canadian Census	Neighbourhood income quintile and rurality[a]	• Contains statistical data on individuals living in Canada.<br>• The RPDB database was linked to the Canadian Census data (2006, 2011 and 2016) using the Postal Code Conversion File (PCCF) to obtain neighbourhood characteristics.
Ontario Marginalization Index (ONMARG)[b]	Material deprivation index quintile	• The Postal Code Conversion File (PCCF) was used to link the RPDB database to the ONMARG database, which is a Canadian Census derived database used to obtain material deprivation index quintiles.<br>• The ONMARG database is available for years 2001, 2006, 2011 and 2016.
Ontario Health Insurance Plan (OHIP) Claims Database	Outpatient physician visits for:<br>• a sexual assault examination[c]<br>• parent child problems<br>• mental illness[c]<br>• marital problems or divorce[d]	• In Ontario, residents receive publicly funded hospital and physician services through the single payer Ontario Health Insurance Plan (OHIP).<br>• The OHIP Claims database contains most claims (fee and associated diagnostic codes) paid for by OHIP and covers all healthcare professionals who can bill under OHIP,<br>• In Ontario, approximately 95% of specialists and 50% of family physicians receive most of their income from fee-for-service (FFS). However, all physicians (except a small percentage of family physicians who work in Community Health Centres) must submit shadow billings for services provided in a non-FFS format. This helps to ensure that the OHIP Claims database more accurately reflects the utilization of physician services in Ontario.<br>• Data have been collected and reported since 1991.<br>• Quality control checks are completed on the data available within the OHIP Claims database.
National Ambulatory Care Reporting System (NACRS)	Emergency department visits for:<br>• physical assault[c]<br>• sexual assault[c]<br>• neglect or abandonment, psychological abuse, and other maltreatment<br>• mental illness[c]	• Large health administrative database in Canada which captures information on all facility and certain community based ambulatory care visits including day surgery as well as emergency department (ED) visits.<br>• The NACRS database contains demographic, clinical, administrative, financial, and service-specific data elements.<br>• All institutions submit these data elements every fiscal year since 2000.<br>• Pertinent to the current study, a data quality study conducted in 2004-2005 showed that over 80% of the mental and behavioural health codes as well as over 70% of the injury and external cause of morbidity/mortality codes were an exact match compared to the re-abstracted data (less than 5% of the re-abstracted data elements were assigned to a completely different International Classification of Diseases 10th Revision (ICD-10-CA) chapter).[e]

© 2023 Archambault É et al. *JAMA Network Open.*

Discharge Abstract Database (DAD)	Hospitalizations for:<br>• physical assault[c]<br>• sexual assault[c]<br>• neglect or abandonment, psychological abuse, and other maltreatment<br>• mental illness[c]	• Large Canadian health administrative database which contains patient-level data for all acute, rehabilitation, chronic and day surgery facilities in Ontario, which includes demographic, clinical, and administrative data elements, as well as information on length of stay and resource consumption.<br>• Data have been collected and reported since 1988.<br>• Re-abstraction has shown few discrepancies in the non-medical data. In Ontario, approximately 86% of diagnosis codes were an exact match compared to the re-abstracted data during the 2002-2003 and 2003-2004 fiscal years (less than 2% of the re-abstracted diagnosis codes were assigned to a completely different ICD-10-CA chapter). More specifically, there was a 12.5% and 42.4% discrepancy rate for injuries (elbow, forearm, hip and thigh injuries were selected to conduct this analysis) and a 17.4% and 20.9% discrepancy rate for all mental health disorders in 2002-2003 and 2003-2004, respectively.[f]
Ontario Mental Health Reporting System (OMHRS)	Hospitalizations for mental illness[g]	• Collects and reports data since 2005 on patients in adult (≥16 years old) designated mental health facilities, including General, Provincial Psychiatric and Specialty Psychiatric facilities.
MOMBABY Database	Maternal age at delivery	• ICES-derived database that links newborns delivered in Ontario hospitals to their mothers.<br>• The MOMBABY database contains all births from 1988 to 2022.<br>• Mother-child pairs are currently linked using the Maternal-Newborn Matching number, but prior to 2002-2003, linkage was done probabilistically. This probabilistic algorithm was shown to have a sensitivity of 96.1% and a specificity of 99.2% for determining mother-child pairs. In 2018, less than 1% of the matches were considered suspicious (i.e., possible error in linkage between mothers and their newborns).[h]
Immigration, Refugees and Citizenship Canada (IRCC) Permanent Resident Database (CIC Database)	Immigration status	• Contains demographic information on individuals who immigrated into Ontario.<br>• Data elements are available since 1985.<br>• Individuals in the IRCC Permanent Resident database obtain an ICES Key Number (IKN) through deterministic and probabilistic linkage with the RPDB database. The overall linkage rate from 1985 to 2012 was 86.4% (migrants from East Asia had the lowest overall linkage rate at 78%).[i]
Office of the Registrar General's Vital Statistics – Death Database (ORGD Database)	Completed suicide	• Contains information on all deaths registered in Ontario.<br>• Data have been collected and reported since 1990.<br>• Cause of death information (available until December 2017 for our study) lags other variables by approximately 2 years.<br>• Individuals in the ORGD database are assigned an ICES Key Number (IKN) through deterministic and probabilistic linkage with the RPDB database. The overall linkage rate from 1990 to 2013 for the ORGD database was 96.2%. Pertinent to the current study, individuals who died from injuries or poisoning (including suicide) had an overall linkage rate of 93.8% and there was a sensitivity of 97% between coroner classification of suicide deaths and vital statistics.[j,k]

© 2023 Archambault É et al. *JAMA Network Open.*

a. Neighbourhood income quintile and rurality were obtained using the Postal Code Conversion File (PCCF) and an individual’s postal code contained in the RPDB database
b. Canadian Census derived database
c. For children and linked mothers
d. Maternal variable only
e. Canadian Institute for Health Information (CIHI). CIHI Data Quality Study of Ontario Emergency Department Visits for 2004-2005 - Main Study Findings. In. Vol II2008
f. Health Results Team for Information Management. Reabstraction Study of the Ontario Case-costing Facilities for Fiscal Years 2002/2003 and 2003/2004. In:2005
g. For children > 16 years old and linked mothers
h. ICES. Ontario Mother-Baby linked dataset (MOMBABY). ICES intranet. Accessed. November 11, 2020
i. Chiu M, Lebenbaum M, Lam K, et al. Describing the linkages of the immigration, refugees and citizenship Canada permanent resident data and vital statistics death registry to Ontario's administrative health database. *BMC Med Inform Decis Mak.* 2016;16(1):135
j. ICES. Vital Statistics - Death, Office of the Registrar General- Deaths (ORGD). ICES intranet. Accessed. November 11, 2020
k. Gatov E, Kurdyak P, Sinyor M, Holder L, Schaffer A. Comparison of Vital Statistics Definitions of Suicide against a Coroner Reference Standard: A Population-Based Linkage Study. Can J Psychiatry. 2018;63(3):152-60

© 2023 Archambault É et al. *JAMA Network Open.*

eTable 2. *ICD-10-CA* Codes Used to Define Physical Assault

Type of service use	ICD-10-CA code	Description
ED visit[a] or Hospitalization[b]	R456	Physical violence
	T741	Physical abuse
	X85-X99 and Y00-Y04	Assault
	Y871	Sequelae of assault
	Z0451	Examination and observation following alleged child sexual and physical abuse
	Z616	Problems related to alleged physical abuse of child

a. ICD-10-CA codes abstracted from the NACRS database
b. ICD-10-CA codes abstracted from the DAD database

© 2023 Archambault É et al. *JAMA Network Open.*

eTable 3. *ICD-10-CA* and OHIP Billing Codes Used to Define Sexual Assault and Other Forms of Child Maltreatment

Type of service use	ICD-10-CA code	Description
ED visit[a] or Hospitalization[b]	T742 and Y05	Sexual assault
	T740 and Y06	Neglect or abandonment
	T743	Psychological abuse
	T748	Other maltreatment syndromes
	T749	Maltreatment syndrome unspecified
	Y07	Other maltreatment
Outpatient physician visit[c]	K018 and K021	Sexual assault examination

a. ICD-10-CA codes abstracted from the NACRS database
b. ICD-10-CA codes abstracted from the DAD database
c. OHIP billing codes abstracted form the OHIP Claims database

© 2023 Archambault É et al. *JAMA Network Open.*

eTable 4. *ICD-10-CA* and OHIP Billing Codes Used to Define Maternal Variables

Mental variables	ICD-9-CM codes[a]	ICD-10-CA codes[b]	OHIP codes[c]
Maternal active mental illness	Any OMHRS code except:<br>- 290.x and 290.x (prior to 2016-2017)<br>- 290.x, 294.0x-294.7x, 294.9x (after 2016-2017)	DX10CODE1= F06-F99 or DX10CODE2-DX10CODE10 = X60-X84, Y10-Y19, Y28 when DX10CODE1 not equal to F06-F99	295, 296, 297, 298, 300, 301, 302, 303, 304, 306, 309, 311, 897, 898, 899, 900, 901, 902, 904, 905, 906, 909
Maternal exposure to violence resulting in the assessment by a healthcare provider	N/A	R456, T741, T742, X85-Y09, Y871, Z0450	K018
Marital problems or divorce	N/A	N/A	898, 901

a. ICD-9-CM codes abstracted from the OMHRS database
b. ICD-10-CA codes abstracted from the NACRS and DAD databases
c. OHIP codes abstracted from the OHIP Claims database

© 2023 Archambault É et al. *JAMA Network Open.*

eTable 5. Mental Illness Codes and Diagnostic Categories Used to Define Primary and Secondary Mental Health Outcomes

Mental illness diagnostic category	ICD-9-CM codes[a]	ICD-10-CA codes[b]	OHIP codes[c]
Incident mental illness	DXCODE1 291.x-293.x, 295.x-319.x (including missing diagnoses; excluding 290.x OR 294.x OR 293.0 = F05 OR 299.x).	DX10CODE1= F06-F99 (excluding F84) or DX10CODE1-DX10CODE10 = X60-X84	295, 296, 297, 298, 299, 300, 301, 302, 306, 307, 309, 311, 313, 314, 315, 291, 292, 303, 304, 897, 899, 902, 904, 905, 906, 909
Psychotic disorders	293.81/82, 295.x (all 295 codes), 297.x (all 297 codes), 298.x (all 298 codes). Provisional=2	F06.0-2, F20, F22-F29, F53.1	295, 296, 297, 298, 299
Non-psychotic disorders	*Mood disorders:* 293.83, 296.x (all 296 codes), 300.4x, 301.13, 311.x, 625.4. Provisional=3, 4 *Anxiety disorders:* 293.84, 300, 300.0x, 300.2x, 309.21, 313.23. Provisional=5 *Trauma/stressor related disorders:* 308.3x, 309, 309.0x, 309.24, 309.28, 309.3x, 309.4x, 309.81, 309.89, 309.9x, 313.89. Provisional=7 *OCD and related disorders:* 300.3x, 300.7x, 698.4x. Provisional=6 *Personality disorders:* 301, 301.0x, 301.2x, 301.4x, 301.5x, 301.6x, 301.7x, 301.81-3, 301.89, 301.9x 310.1. Provisional=18	*Mood disorders:* F06.3, F30.x-F34.x, F38.x, F39.x, F53.0 *Anxiety disorders:* F06.4, F40, F41, F93.0-2, F94.0 *Trauma/stressor related disorders:* F43.x, F94.1, F94.2 *OCD and related disorders:* F42.x, F45.2, F63.3 *Personality disorders:* F07.0, F21, F60, F51, F62, F68, F69	300 (Anxiety neurosis, hysteria, neurasthenia, obsessive-compulsive neurosis, reactive depression); 301 (Personality disorders); 309 (Adjustment reaction); 311 (Depressive disorder)
Substance use disorders	291.x (all 291 codes), 292.x (all 292 codes), 303.x (all 303 codes), 304.x (all 304 codes), 305.x. Provisional=16	F10-19, F55, F63.0	291, 292, 303, 304
Select childhood behaviour disorders	314.xx, 312.xx, 313.81	F90, F91	313, 314
Intentional self-injury	N/A	DX10CODE1-DX10CODE10 = X60-X84	N/A

© 2023 Archambault É et al. *JAMA Network Open.*

Mental illness diagnostic category	ICD-9-CM codes[a]	ICD-10-CA codes[b]	OHIP codes[c]
Other disorders	291.x-293.x, 295.x-319.x (including missing diagnoses) not included in above categories (excluding 290.x OR 294.x OR 293.0 = F05 OR 299.x)	F06-F99 not included in above categories (excluding F84)	302 (Sexual deviations); 306 (Psychosomatic illness); 307 (Habit spasms, tics, stuttering, tension headaches, anorexia nervosa, sleep disorders, enuresis); 315 (Specific delays in development (e.g., dyslexia, dyslalia, motor retardation); 897 (economic problems); 899 (Parent-child problems); 902 (Educational problems); 904 (Social maladjustment); 905 (Occupational problems); 906 (Legal problems); 909 (Other problems of social adjustment)

a. ICD-9-CM codes abstracted from the OMHRS database
b. ICD-10-CA codes abstracted from the DAD and NACRS databases
c. OHIP codes abstracted from the OHIP Claims database. Any outpatient OHIP visit/consult to a psychiatrist; any outpatient OHIP visit/consult to a family physician with a mental health diagnostic code; any outpatient OHIP visit/consult to a pediatrician with mental health diagnostic code, or any OHIP visit/consult to a pediatrician with the following service codes (K122, K123, K704) and a mental health diagnostic code.

© 2023 Archambault É et al. *JAMA Network Open.*

eTable 6. Baseline Characteristics of Childhood Survivors of Physical Assault and Matched Unexposed Children (Cohort Restricted to Children Linked to Their Mothers via the MOMBABY Database)

All values represent n (%) unless otherwise specified.

Baseline characteristic	Unexposed to assault N=17,680	Exposed to assault N=4,420	Standardized difference[a]
Age (at index), mean (SD)	6.54 ± 4.6	6.54 ± 4.6	0.00
Sex (male)	7,841 (44.3)	2,359 (53.4)	0.18
Material Deprivation Index Quintile (1)	3,293 (18.6)	511 (11.6)	0.20
Material Deprivation Index Quintile (2)	3,327 (18.8)	597 (13.5)	0.14
Material Deprivation Index Quintile (3)	3,214 (18.2)	775 (17.5)	0.02
Material Deprivation Index Quintile (4)	3,376 (19.1)	811 (18.3)	0.02
Material Deprivation Index Quintile (5)	3,740 (21.2)	1,429 (32.3)	0.25
Material Deprivation Index Quintile (missing)	730 (4.1)	297 (6.7)	0.11
Neighbourhood Income Quintile (1)	3,206 (18.1)	1,383 (31.3)	0.31
Neighbourhood Income Quintile (2)	3,215 (18.2)	952 (21.5)	0.08
Neighbourhood Income Quintile (3)	3,584 (20.3)	783 (17.7)	0.07
Neighbourhood Income Quintile (4)	3,851 (21.8)	693 (15.7)	0.16
Neighbourhood Income Quintile (5)	3,746 (21.2)	548 (12.4)	0.24
Neighbourhood Income Quintile (missing)	78 (0.4)	61 (1.4)	0.10
Rurality (rural)	1,092 (6.2)	1,003 (22.7)	0.48
Sexual assault	‡	13 (0.3)	0.07
Maltreatment other	‡	9 (0.2)	0.06
Parent child problems	‡	25 (0.6)	0.11
Other forms of maltreatment combined[b]	‡	46 (1.0)	0.14
Maternal active mental illness	4,196 (23.7)	1,894 (42.9)	0.41
Marital problem or divorce	373 (2.1)	186 (4.2)	0.12
Maternal violence/assault	100 (0.6)	250 (5.7)	0.30
Maternal age < 19 at birth	423 (2.4)	760 (17.2)	0.51

[a]A standardized difference > 0.10 is considered significant
[b]Composite variable composed of sexual assault, maltreatment other, and parent child problems
‡ Presence of small cells omitted from table to reduce the risk of re-identification

© 2023 Archambault É et al. *JAMA Network Open.*

eTable 7. Association Between Childhood Physical Assault Diagnosed in an Acute Care Setting and Incident Health Record Diagnoses of Mental Illness in the Restricted Study Cohort[a]: Incidence Rate as Well as Crude and Adjusted Hazard Ratios[b]

	n	Patient-Years	n (%) with mental illness	IR per 1000 Person-Years (95% CI)	cHR (95% CI)	aHR (95% CI)
Childhood survivors of physical assault	4,420	32,867	1,747 (39.5)	53.15 (50.69 - 55.71)	1.87 (1.76 - 1.98)	1.75 (1.63 - 1.88)*
Unexposed children	17,680	132,615	4,318 (24.4)	32.56 (31.60 - 33.55)	Ref	Ref
**Partitioning of the time axis[c]**						
**0 - <1 year following physical assault**						
Childhood survivors of physical assault	4,420	4,403	369 (8.3)	83.81 (75.48 - 92.82)	3.14 (2.74 - 3.60)	2.94 (2.50 - 3.46)**
Unexposed children	17,680	17,577	488 (2.8)	27.76 (25.35 - 30.34)	Ref	Ref
**1 - <4 years following physical assault**						
Childhood survivors of physical assault	4,023	11,934	780 (19.4)	65.36 (60.85 - 70.11)	1.91 (1.75 - 2.08)	1.75 (1.58 - 1.94)**
Unexposed children	17,019	50,744	1,775 (10.4)	34.98 (33.37 - 36.65)	Ref	Ref
**≥4 years following physical assault**						
Childhood survivors of physical assault	3,169	11,744	598 (18.9)	50.92 (46.92 - 55.17)	1.43 (1.30 - 1.58)	1.37 (1.22 - 1.53)*
Unexposed children	15,077	54,774	2,055 (13.6)	37.52 (35.91 - 39.18)	Ref	Ref

IR = incidence rate, CI = confidence interval, cHR = crude hazard ratio, aHR = adjusted hazard ratio

[a]Cohort restricted to children linked to their mothers via the MOMBABY database

[b]Hazard ratio adjusted for sex, rurality, material deprivation index quintile, other forms of child maltreatment and maternal variables

[c]Time following physical assault diagnosis

aHR*: Main exposure adjusted for sex, time*sex, rurality, material deprivation index quintile, other forms of child maltreatment and maternal variables

aHR**: Main exposure adjusted for sex, rurality, material deprivation index quintile, other forms of child maltreatment and maternal variables

Maternal variables include active maternal mental illness, maternal assault, maternal age at birth and maternal divorce/marital problems

© 2023 Archambault É et al. *JAMA Network Open.*

eTable 8. Mental Health Outcomes in Childhood Survivors of Physical Assault and Matched Unexposed Children (Cohort Restricted to Children Linked to Their Mothers via the MOMBABY Database)

All values represent n (%) unless otherwise specified.

Mental health outcome	Unexposed to assault N=17,680	Exposed to assault N=4,420	Standardized difference[a]
Incident mental illness	4,318 (24.4)	1,747 (39.5)	0.33
**Acuity of mental illness[b]**			
Non-acute outpatient mental illness diagnosis	3,844 (21.7)	1,235 (27.9)	0.14
Acute care[c] mental illness diagnosis	481 (2.7)	600 (13.6)	0.40
**Individual components of the composite mental illness variable[d]**			
Non-acute outpatient visit	4,214 (23.8)	1,621 (36.7)	0.28
Intentional self-injury	82 (0.5)	93 (2.1)	0.15
Emergency department visit	453 (2.6)	567 (12.8)	0.39
Inpatient admission	142 (0.8)	189 (4.3)	0.22
Suicide	‡	‡	0.02
**Incident mental illness diagnostic category**			
Intentional self-injury	28 (0.2)	30 (0.7)	0.08
Non-psychotic disorders	1,908 (10.8)	714 (16.2)	0.16
Select childhood behavior disorders[e]	995 (5.6)	484 (11.0)	0.19
Substance use disorders	78 (0.4)	83 (1.9)	0.13
Psychotic disorders	52 (0.3)	22 (0.5)	0.03
Other disorders[f]	1,255 (7.1)	413 (9.3)	0.08

[a]A standardized difference > 0.10 is considered significant
[b]Acuity of mental illness at the time of incident mental illness diagnosis (first contact with the healthcare system)
[c]Acute care = emergency department visit, inpatient admission or suicide
[d]Prevalence throughout the observation period
[e]Attention deficit/hyperactivity disorder, opposional defiant disorder and conduct disorder
[f]Include eating, sleep, sexual, developmental, intellectual, gender identity and tic disorders
‡ Presence of small cells omitted from table to reduce the risk of reidentification

© 2023 Archambault É et al. *JAMA Network Open.*

eTable 9. Association Between Childhood Physical Assault Diagnosed in an Acute Care Setting and the Different Mental Illness Diagnostic Categories in the Restricted Study Cohort[a]: Adjusted Hazard Ratios[b]

Mental illness diagnostic category	aHR (95% CI) 0 - <1 year following physical assault[c]	aHR (95% CI) 1 - <4 years following physical assault[c]	aHR (95% CI) ≥4 years following physical assault[c]
Psychotic disorders	2.41 (1.16 - 4.50)		
Non-psychotic disorders	2.76 (2.07 - 3.69)	1.85 (1.58 - 2.17)	1.26 (1.08 - 1.47)
Select childhood behaviour disorders[d]	3.72 (2.69 - 5.15)	1.96 (1.60- 2.41)	4 year - <6 years: 1.69 (1.26 - 2.26)<br>≥6 years: 1.23 (0.84 - 1.79)
Intentional self injury	5.57 (3.54 - 8.77)		
Substance use disorders	15.33 (6.24 - 37.66)[e,f]	3.77 (1.98 - 7.18)[g]	3.41 (1.81 - 6.44)
Other disorders[h]	2.54 (1.93 - 3.36)	1.26 (1.04 - 1.54)	0.80 (0.61 - 1.04)

CI = confidence interval, aHR = adjusted hazard ratio
[a]Cohort restricted to children linked to their mothers via the MOMBABY database
[b]Hazard ratio adjusted for sex, rurality, material deprivation index quintile, other forms of child maltreatment and maternal variables
[c]Partitioning of the time axis: time following physical assault diagnosis
[d]Attention deficit/hyperactivity disorder, opposional defiant disorder and conduct disorder
[e]Crude hazard ratio presented here because there were few outcomes in that time interval
[f]Up to 17 months due to non-proportional hazards
[g]17 months to 4 years due to non-proportional hazards
[h]Include eating, sleep, sexual, developmental, intellectual, gender identity and tic disorders

© 2023 Archambault É et al. *JAMA Network Open.*
